# Editorial: The blood-brain barrier in brain tumors: molecular mechanisms and therapeutic strategies

**DOI:** 10.3389/fneur.2023.1225594

**Published:** 2023-07-13

**Authors:** Lei Zhang, Anna Dimberg, Javad Rasouli

**Affiliations:** ^1^China-Sweden International Joint Research Center for Brain Diseases, Key Laboratory of Ministry of Education for Medicinal Plant Resource and Natural Pharmaceutical Chemistry, National Engineering Laboratory for Resource Developing of Endangered Chinese Crude Drugs in Northwest of China, College of Life Sciences, Shaanxi Normal University, Xi'an, China; ^2^Science for Life Laboratory, Department of Immunology, Genetics and Pathology, Uppsala University, Uppsala, Sweden; ^3^Rudbeck Laboratory, Uppsala University, Uppsala, Sweden; ^4^Spark Therapeutics Inc, Philadelphia, PA, United States

**Keywords:** blood-brain barrier, glioma, drug delivery, focus ultrasound, blood-tumor barrier

The blood-brain barrier (BBB) is a specialization of brain endothelial cells (ECs) that hinders the effective delivery of drugs into the brain by establishing EC junctions, sealing EC-EC contacts, pumping drugs out by active transport, and suppressing transcytosis (1, 2). In brain tumors, blood vessels are abnormal with a heterogeneous and partially intact BBB, known as the blood-tumor barrier (BTB) (1, 3). The extremely limited systemic therapeutic options after surgery and radiotherapy are one of the culprits for the dismal prognosis in brain tumor patients. Brain tumors can only be cured if the tumor cells hidden behind the BBB are adequately treated (4–6). This Editorial will review current issues in addressing the BBB and, more specifically, introduce four manuscripts included in this Research Topic.

## Opening of the BBB by the FUS technique

The potential of opening the BBB using focused ultrasound (FUS) has received intense interest as a tool to aid the delivery of therapeutic drugs into lesions of the central nervous system (CNS). FUS is a non-invasive therapeutic tool that can induce both thermal ablation and a transient opening of the BBB/BTB. FUS utilizes a special concave transducer, lens, or phased array to converge ultrasound waves into a millimeter-sized precise focal region. BBB/BTB can be transiently opened for access to therapeutics in the brain using FUS targeting intravenously administered microbubbles (MBs). The MBs will oscillate with expansions and contractions during the compression and rarefaction phases of the ultrasound pressure wave in the vasculature. The constantly changing morphology and oscillation of MBs result in shear stress on endothelial cells, which sufficiently breaks tightly sealed junctions in the BBB/BTB by mechanical forces. Mungur et al. provide a systematic review of the opening of the BBB by FUS to enhance drug delivery in glioblastoma (GBM) treatment, together with a comprehensive summary of FUS application in preclinical studies with animal GBM models and recent clinical trials in GBM patients. Thombre et al. reviewed currently used methods for FUS-mediated BBB openings in rodent GBM models and proposed standard parameters for BBB opening with high efficiency and low side effects.

## The role of nanodelivery systems in brain therapies

Nanodelivery systems are increasingly being developed using various strategies to enhance BBB penetration and accumulation in the brain parenchyma. Extensive efforts have been made to successfully improve the transport of nanomaterials across the BBB through, for example, receptor-mediated transcytosis and shutter peptide-mediated BBB-crossing. In addition, changing characteristic properties of nanomaterials, including particle size, composition, hydrophobicity, charge, and dissociation degree, provides a broad space for researchers to modify the system, developing more promising strategies for nanomaterial-based BBB crossing. Liu et al. summarized available nanodelivery systems for non-permeable phytochemicals to the CNS, boosting novel applications of nanodelivery for brain diseases.

Semyachkina-Glushkovskaya et al. provide evidence that music induces BBB opening via the brain drainage system, improving the therapeutic effects of bevacizumab in a rat GBM model. This study sheds new light on the application of music therapy for the treatment of malignant brain tumors.

Altogether, this Research Topic presents recent advances in strategies to improve drug delivery across the BBB, facilitating the design and application of novel therapeutic strategies for GBM treatment.

## Author contributions

LZ contributed to organizing the topic review and wrote the manuscript. AD and JR polished and revised the editorial. All authors listed have made a substantial, direct, and intellectual contribution to the work and approved it for publication.
